# Meeting breastfeeding intentions differ by race/ethnicity, Infant and Toddler Feeding Practices Study‐2

**DOI:** 10.1111/mcn.13093

**Published:** 2020-10-01

**Authors:** Heather C. Hamner, Jennifer L. Beauregard, Ruowei Li, Jennifer M. Nelson, Cria G. Perrine

**Affiliations:** ^1^ Division of Nutrition, Physical Activity, and Obesity, National Center for Chronic Disease Prevention and Health Promotion Centers for Disease Control and Prevention (CDC) Atlanta Georgia USA; ^2^ United States Public Health Service Commissioned Corps Rockville Maryland USA; ^3^ Center for Surveillance, Epidemiology, and Laboratory Services, Centers for Disease Control and Prevention Epidemic Intelligence Service Atlanta Georgia USA

**Keywords:** breastfeeding, breastfeeding intentions, low‐income women, racial/ethnic disparities, WIC

## Abstract

Prenatal breastfeeding intentions impact breastfeeding practices. Racial/ethnic disparities exist in breastfeeding rates; it is unknown if prenatal intentions and meeting intentions differ by race/ethnicity. A longitudinal cohort of USDA's Special Supplemental Nutrition Program for Women, Infants, and Children (WIC) which enrolled participants beginning in 2013 were used to estimate prenatal intentions for breastfeeding initiation, exclusive breast milk feeds at 1 and 3 months by race/ethnicity (*n* = 2070). Meeting intentions were determined by reported breast milk consumption at birth, 1 month and 3 months. Multivariable logistic regression was used to determine the association of race/ethnicity with meeting intentions. There were no differences in prenatal breastfeeding intentions between non‐Hispanic White and non‐Hispanic Black women (initiation: 86.9% and 87.2%; Month 1: 52.3% and 48.3%; Month 3: 43.8% and 40.9%; respectively), but a higher percentage of Hispanic women intended to breastfeed at all time points (95.5%, 68.3% and 56.4%; respectively, *P* < 0.05). Among women who intended to breastfeed at Month 1, non‐Hispanic Black and Hispanic women had significantly lower odds of meeting intentions compared with non‐Hispanic White women after adjusting for covariates (aORs: 0.63 [95% CI: 0.41, 0.98]; 0.64 [95% CI: 0.44, 0.92], respectively). Similar findings were seen for Month 3. Despite no differences in breastfeeding intentions, non‐Hispanic Black women were less likely to meet their breastfeeding intentions than non‐Hispanic White women. Hispanic women were more likely to intend to breastfeed yet were less likely to meet their intentions. This suggests that non‐Hispanic Black and Hispanic women face challenges to meeting their longer breastfeeding intentions. Understanding how racism, bias and discrimination contribute to women not meeting their breastfeeding intentions may help efforts to reduce breastfeeding disparities.

**Key messages**

- Among a longitudinal cohort of WIC participants, no differences in prenatal breastfeeding intentions existed between non‐Hispanic Black and non‐Hispanic White women.
- Meeting breastfeeding intentions differed by race/ethnicity.
- Hispanic and non‐Hispanic Black women were less likely to meet their longer breastfeeding intentions than non‐Hispanic White women.
- Understanding how racism, bias and discrimination contribute to women not meeting their breastfeeding intentions may help efforts to reduce breastfeeding disparities.

## 1 INTRODUCTION

Breast milk is the optimal source of nutrition for most infants (Section on Breastfeeding, 2012). The American Academy of Pediatrics recommends that infants receive only breast milk for about the first 6 months of life, followed by complementary food introduction with continued breastfeeding for as long as mother and baby desire (Section on Breastfeeding, 2012). Although breastfeeding rates have increased overall in the United States for the last several decades, racial disparities persist (Centers for Disease Control and Prevention, 2019; Grummer‐Strawn & Shealy, 2009). For example among children born in 2016, 74.0% of non‐Hispanic Black infants have ever breastfed compared with 86.6% of non‐Hispanic White infants (Centers for Disease Control and Prevention, 2019). Similarly, non‐Hispanic Black infants have lower exclusive breastfeeding rates than non‐Hispanic White infants at both 3 and 6 months (Centers for Disease Control and Prevention, 2019).

There are many factors the can impact breastfeeding intentions, initiation and duration and these factors may differ by race/ethnicity (Asiodu, Waters, Dailey, & Lyndon, 2017; Hinson, Skinner, Lich, & Spatz, 2018; Spencer & Grassley, 2013). Historically, advice and/or support for breastfeeding from the US Department of Agriculture's (USDA's) Special Supplemental Nutrition Program for Women, Infants, and Children (WIC) programme has differed between Black and White women with Black women being more likely to receive support for bottle feeding and less likely to receive advice on breastfeeding (Beal, Kuhlthau, & Perrin, 2003). More recently, African American women who breastfed reported that WIC peer counsellors or participation in a WIC peer breastfeeding support group played a positive role in helping them breastfeed (Gross, Davis, Anderson, Hall, & Hilyard, 2017; Mickens, Modeste, Montgomery, & Taylor, 2009; Robinson, VandeVusse, & Foster, 2016). The decision to breastfeed is often made prenatally (Gurka et al., 2014) and has been found to be a strong predictor of actual breastfeeding practice (Donath, Amir, & The ALSPAC Study Team, 2003). Limited data exist on whether prenatal breastfeeding intentions differ by race/ethnicity and whether or not women are able to achieve these intentions. The objective of our study is to describe women's prenatal breastfeeding intentions by race/ethnicity and assess the impact of race/ethnicity in meeting these intentions.

## 2 METHODS

USDA's WIC programme conducted a longitudinal cohort study of women and their children enrolled in WIC entitled the WIC Infant and Toddler Feeding Practices Study 2 (ITFPS‐2). This longitudinal cohort study is designed to provide information on feeding practices and nutrition outcomes among women and children enrolled in WIC (May et al., 2017). Sampling for the study was done using a two stage stratified approach as described in the WIC ITFPS‐2: Infant Report (May et al., 2017). Briefly, the study enrolled participants in 2013 from 80 WIC sites across 27 states. WIC sites that were projected to enrol ≥30 participants per month were eligible to participate. These sites represented 37% of WIC sites and 87% of WIC participants (May et al., 2017). Women were invited to participate if they were ≥16 years of age, spoke English or Spanish and were either pregnant or their infant was less than two and half months old (May et al., 2017). Women completed an in‐person screener to determine eligibility. Overall, a total of 4489 women were screened and eligible to complete the study and 4367 enrolled (97.3%). Of these, 2649 enrolled prenatally and completed a prenatal interview; an additional 1400 women enrolled after birth and completed the 1‐ or 3‐month interview; and 318 did not complete either a prenatal, Month 1 or Month 3 interview after enrollment. Prenatal interviews and all follow‐up surveys were completed via telephone in English or Spanish. A total of 16 follow‐up surveys were completed from birth to 60 months at approximately 2‐ to 6‐month intervals (i.e., Months 1, 3, 5, 7, 11, 13, 15, 18, 24, 30, 36, 42, 48, 54, 60 and 72). Prenatal interviews were completed within 60 days of enrollment and follow‐up surveys were open for 4 weeks (i.e., starting 14 days before through 14 days after the participant reached the age of the monthly survey; meaning this could be as early as 2 weeks of age or as late as 6 weeks of age for the Month 1 survey). Women provided written informed consent and were provided incentives for enrolling and for each survey completed (May et al., 2017). We use three survey time points for this analysis: the prenatal, Month 1 and Month 3 interviews. For this analysis, no infants enrolled after birth were included because data were needed on prenatal intentions. Response rates for these interviews ranged from 87.7% to 92.3% (May et al., 2017). Detailed study methodology has been published (May et al., 2017).

### 2.1 Breastfeeding intentions and meeting intentions

Prenatal breastfeeding intentions were assessed using a 5‐point Likert scale to three questions: (1) ‘I am planning to breastfeed my baby or at least try’, (2) ‘When my baby is one month old, I will be breastfeeding without using any formula or other milk’, and (3) ‘When my baby is three months old, I will be breastfeeding without using any formula or other milk’. Women who reported strongly agree/agree to the questions were categorized as intending to initiate breastfeeding (question one), breastfeed at Month 1 (Question 2) or breastfeed at Month 3 (Question 3).

Meeting breastfeeding intentions were determined only among women who intended to initiate breastfeeding, breastfeed at Month 1 or breastfeed at Month 3. Thus, meeting each of the three breastfeeding intention outcomes were examined independently and had a different denominator (those who disagreed/strongly disagreed or were neutral were not included in the denominator at each intention time point). Meeting breastfeeding initiation intention was defined as reporting that they initiated breastfeeding (May et al., 2017). Breastfeeding initiation was based on indication of breast milk consumption in any of a series of questions 1 assessing the first thing fed, feeding in the hospital, current feeding practices and feeding from a bottle. If mothers did not indicate breast milk consumption, they were classified as not initiating breastfeeding. Meeting intentions to breastfeed at Month 1 or at Month 3 were defined as the infant currently only receiving breast milk (no formula) at the Month 1 interview or at the Month 3 interview, respectively. Not meeting intentions to breastfeed at Month 1 or 3 included infants who were currently (1) receiving both breast milk and infant formula or (2) receiving only infant formula. These were the only three response options available and respondents may not have considered any other food or beverage a child was consuming when answering. Therefore, this categorization cannot be interpreted as a true reflection of exclusive breastfeeding because they do not account for any beverage or food other than breast milk or infant formula that was additionally reported.

### 2.2 Analytic sample

We limited our analyses to women who completed at least 50% of the prenatal interview in either English or Spanish (*n* = 2649). Women were excluded if they did not complete the Month 1 interview (*n* = 414) and the Month 3 interview (*n* = 164) or if they were missing data on the intentions questions (*n* = 1). This left a final sample size of 2070, representing 78% of those who enrolled prenatally.

### 2.3 Covariates

Covariates include maternal age at birth (16–19, 20–25 or ≥26 years), maternal education status (≤high school or >high school), breastfeeding history (any or none), type of delivery (vaginal or caesarean), preterm status (baby born > 3weeks before due date) and maternal race/ethnicity (non‐Hispanic White, non‐Hispanic Black or African American [referred to as non‐Hispanic Black]) and Hispanic. Women of other race/ethnicities are included in all analyses except when stratified by race/ethnicity. Household characteristics included status of mother and father living in the same household (yes/no) and poverty level (≤75%, 76% to 130% and >130% of the 2013 poverty guidelines for annual household income; U.S. Health and Human Services, 2013). Covariates were chosen because of their association with differences in national breastfeeding rates (Centers for Disease Control and Prevention, 2019; Chiang, Sharma, Nelson, Olson, & Perrine, 2019).

### 2.4 Statistical analyses

We estimated the percentage of women's prenatal breastfeeding intentions and the percentage meeting those intentions for initiation and at Month 1 and Month 3, overall and by race/ethnicity. Differences in proportions by race/ethnicity were assessed using *t*‐tests (*P* < 0.05). Multivariable logistic regression was done to assess the association of race/ethnicity with meeting prenatal breastfeeding intentions for (1) initiating breastfeeding, (2) feeding only breast milk at Month 1 and (3) feeding only breast milk at Month 3. Odds ratios (ORs) were adjusted for all covariates.

SPSS Complex Samples version 25.0 (IBM SPSS Inc, Chicago, IL) was used in all analyses to account for the complex sample design. Analyses were weighted using the prenatal interview core weight which adjusts for differential probability of selection and non‐response. Analyses are therefore representative of the WIC population who were enrolled in WIC sites with ≥30 participants/month.

## 3 RESULTS

Table 1 describes the demographic characteristics of the analytic sample. Among all women, 32.1% were non‐Hispanic White, 20.9% were non‐Hispanic Black and 41.4% were Hispanic. Additionally, 49.3% were ≥26 years of age at the time of birth, 62.5% had high school education or less, 50.6% did not have any breastfeeding history, 67.0% had a vaginal delivery and 11.0% had a baby born > 3 weeks before the due date. Over half (55.0%) were living with the father of the baby, and 62.0% were living with a household income ≤ 75% of the poverty guidelines.

**TABLE 1:** Demographic characteristics of mothers and babies enrolled in WIC by maternal race/ethnicity,^a^ USDA's Infant and Toddler Feeding Practices Study 2

	Total population (*n* = 2070)	Non‐Hispanic White (*n* = 665)	Non‐Hispanic Black (*n* = 432)	Hispanic (*n* = 858)
	% (95% confidence interval)			
Maternal characteristics	—	32.1 (30.1, 34.2)	20.9 (19.2, 22.7)	41.4 (39.3, 43.6)
Age at birth				
16–19 years	11.6 (9.2, 14.4)	8.6 (6.3, 11.7)	13.1 (7.4, 22.2)	12.9 (9.6, 17.2)^*^
20–25 years	39.2 (35.9, 42.5)	44.5 (39.4, 49.8)	41.0 (35.3, 47.0)	35.7 (31.2, 40.4)^*^ ^,^ ^**^
≥26 years	49.3 (45.1, 53.5)	46.9 (41.0, 52.8)	45.9 (35.8, 56.4)	51.4 (46.3, 56.4)^**^
Education status				
High school or less	62.5 (59.0, 65.9)	59.1 (54.2, 63.8)	56.6 (49.7, 63.2)	67.9 (62.1, 73.2)^*^ ^,^ ^**^
More than high school	37.5 (34.1, 41.0)	40.9 (36.2, 45.8)	43.4 (36.8, 50.3)	32.1 (26.8, 37.9)^*^ ^,^ ^**^
Breastfeeding history				
No breastfeeding history	50.6 (47.0, 54.3)	52.3 (46.7, 57.8)	60.1 (53.7, 66.2)^*^	45.7 (40.1, 51.5)^*^ ^,^ ^**^
Any breastfeeding history	49.4 (45.7, 53.0)	47.7 (42.2, 53.3)	39.9 (33.8, 46.3)^*^	54.3 (48.5, 59.9)^*^ ^,^ ^**^
Type of delivery				
Vaginal	67.0 (64.7, 69.2)	71.1 (66.9, 75.1)	65.9 (59.3, 71.9)	64.7 (60.8, 68.4)^*^
Caesarean	33.0 (30.8, 35.3)	28.9 (24.9, 33.1)	34.1 (28.1, 40.7)	35.3 (31.6, 39.2)^*^
Preterm status				
Born > 3 weeks before due date	11.0 (9.2, 13.1)	9.7 (7.4, 12.6)	16.5 (12.8, 21.0)^*^	9.0 (7.1, 11.4)
Household characteristics				
Mother and father in household				
Father of baby living with mother	55.0 (50.8, 59.1)	63.4 (57.3, 69.0)	33.2 (25.8, 41.4)^*^	58.1 (53.1, 63.0)^*^ ^,^ ^**^
Father of baby not living with mother	45.0 (40.9, 49.2)	36.6 (31.0, 42.7)	66.8 (58.6, 74.2)^*^	41.9 (37.0, 46.9)^*^ ^,^ ^**^
Poverty level^b^				
≤75% of poverty guideline	62.0 (57.8, 65.9)	50.8 (44.8, 56.9)	69.1 (59.1, 77.5)^*^	66.4 (62.0, 70.5)^*^
76% to 130% of poverty guideline	27.4 (24.6, 30.3)	32.6 (28.9, 36.6)	22.5 (15.9, 30.8)^*^	26.0 (22.1, 30.3)^*^
>130% of poverty guideline	10.7 (8.4, 13.4)	16.5 (12.0, 22.3)	8.4 (4.5, 15.1)^*^	7.6 (5.9, 9.8)^*^ ^,^ ^**^

Abbreviations: USDA, United States Department of Agriculture; WIC, Special Supplemental Nutrition Program for Women, Infants, and Children.

^a^ Race/ethnicity subanalyses are restricted to women who are non‐Hispanic White, non‐Hispanic Black or Hispanic.

^b^ Poverty guideline is defined as the 2013 poverty guidelines (U.S. Health and Human Services, 2013).

^*^ Statistically significantly different than non‐Hispanic White (*t*‐test, *P* < 0.05).

^**^ Statistically significantly different than non‐Hispanic Black (*t*‐test, *P* < 0.05).

There were select demographic characteristics that differed by race/ethnicity. For example, 67.9% of Hispanic women had a high school or less education compared with non‐Hispanic White and non‐Hispanic Black women, 59.1% and 56.6%, respectively (*P* < 0.05). Compared with non‐Hispanic White women, non‐Hispanic Black and Hispanic women were more likely to live in a household with an income ≤ 75% of the poverty guidelines, 50.8%, 69.1% and 66.4%, respectively (*P* < 0.05).

Prenatal breastfeeding intentions are presented in Table 2. Overall, nine in 10 women strongly agreed/agreed with the statement of planning to breastfeed or at least try, with Hispanic women reporting the highest agreement at 95.5% compared with both non‐Hispanic White and non‐Hispanic Black women (86.9% and 87.2%, respectively, *P* < 0.05). Six in 10 women strongly agreed/agreed with the statement that they would be only feeding breast milk when their baby was 1 month old. This percentage was significantly higher among Hispanic (68.3%) compared with non‐Hispanic White (52.3%) and non‐Hispanic Black women (48.3%) (*P* < 0.05). Lastly, five in 10 women strongly agreed/agreed with the statement that they would be only feeding breast milk when their baby was 3 months old. Racial/ethnic differences, similar to Month 1, were observed at Month 3.

**TABLE 2:** Prenatal breastfeeding intentions among women enrolled in WIC by maternal race/ethnicity,^a^ USDA's Infant and Toddler Feeding Practices Study 2

	Total (*n* = 2070)	Non‐Hispanic White (*n* = 665)	Non‐Hispanic Black (*n* = 432)	Hispanic (*n* = 858)
	% (95% confidence interval)^b^			
General breastfeeding intention^c^				
Strongly agree/agree	91.4 (89.3, 93.2)	86.9 (82.1, 90.6)	87.2 (82.1, 91.0)	95.5 (93.7, 96.9)^*^ ^,^ ^**^
Neither agree or disagree	1.6 (1.0, 2.6)	2.5 (1.4, 4.5)	1.3 (0.5, 3.4)	1.2 (0.5, 2.8)^*^
Strongly disagree/disagree	6.9 (5.3, 9.1)	10.5 (7.2, 15.2)	11.5 (7.8, 16.7)	3.2 (2.1, 4.9)^*^ ^,^ ^**^
Intention to only feed breast milk at 1 month of age^d^				
Strongly agree/agree	60.3 (56.4, 64.0)	52.3 (46.2, 58.2)	48.3 (39.7, 57.0)	68.3 (64.2, 72.1)^*^ ^,^ ^**^
Neither agree or disagree	18.1 (15.5, 21.0)	24.3 (20.6, 28.5)	22.3 (18.0, 27.3)	13.1 (9.7, 17.5)^*^ ^,^ ^**^
Strongly disagree/disagree	21.7 (19.1, 24.5)	23.4 (18.6, 29.1)	29.4 (23.5, 36.1)	18.6 (15.5, 22.0)^*^ ^,^ ^**^
Intention to only feed breast milk at 3 months of age^e^				
Strongly agree/agree	50.4 (46.7, 54.0)	43.8 (38.2, 49.5)	40.9 (32.8, 49.6)	56.4 (52.4, 60.2)^*^ ^,^ ^**^
Neither agree or disagree	20.4 (18.1, 22.9)	26.9 (23.0, 31.1)	23.4 (18.8, 28.7)	15.8 (13.4, 18.4)^*^ ^,^ ^**^
Strongly disagree/disagree	29.3 (26.2, 32.5)	29.3 (24.6, 34.6)	35.7 (27.7, 44.7)	27.9 (24.2, 31.9)^**^

Abbreviations: USDA, United States Department of Agriculture; WIC, Special Supplemental Nutrition Program for Women, Infants, and Children.

^a^ Race/ethnicity subanalyses are restricted to women who are non‐Hispanic White, non‐Hispanic Black or Hispanic.

^b^ Estimates do not include a response of Do not Know; for general breastfeeding intention (*n* = 1), for intention at 1 month (*n* = 26) and for intention at 3 months (*n* = 32).

^c^ Responses to the question, ‘I am planning to breastfeed my baby or at least try’.

^d^ Responses to the question, ‘When my baby is one month old, I will be breastfeeding without using any formula or other milk’.

^e^ Responses to the question, ‘When my baby is three months old, I will be breastfeeding without using any formula or other milk’.

^*^ Indicates statistically significantly different than non‐Hispanic White (*t*‐test, *P* < 0.05).

^**^ Indicates statistically significantly different than non‐Hispanic Black (*t*‐test, *P* < 0.05).

Among women who intended to breastfeed at each of the three time points, Table 3 provides the percentage of women who met or did not meet their intentions. Approximately nine in 10 women (90.6%) met their intention of initiating breastfeeding. A significantly higher percentage of Hispanic women were able to meet their initiation intention as compared with non‐Hispanic Black and non‐Hispanic White women (95.3%, 85.4% and 84.4%, respectively, *P* < 0.05).

**TABLE 3:** Percentage of women enrolled in WIC who strongly agreed/agreed with statement on breastfeeding intentions during pregnancy and current feeding practice by maternal race/ethnicity,^a^ USDA's Infant and Toddler Feeding Practices Study 2

	Intended to try breastfeeding			Intended to feed only breast milk at 1 month				Intended to feed only breast milk at 3 months			
	Total	Met intention	Did not meet intention	Total	Met intention	Did not meet intention		Total	Met intention	Did not meet intention	
		Initiated breastfeeding^b^	Did not initiate breastfeeding^c^		Only breast milk^d^	Breast milk and infant formula^e^	Only infant formula^f^		Only breast milk^d^	Breast milk and infant formula^e^	Only infant formula^f^
	*n*	% (95% confidence interval)		*n*	% (95% confidence interval)^g^			*n*	% (95% confidence interval)		
Total	1877	90.6 (88.3, 92.5)	9.4 (7.5, 11.7)	1226	45.2 (41.1, 49.5)	34.8 (31.6, 38.2)	19.9 (17.3, 22.9)	1035	32.9 (28.2, 38.0)	24.9 (20.9, 29.3)	42.2 (38.0, 46.5)
Non‐Hispanic White	571	84.4 (79.3, 88.5)	15.6 (11.5, 20.7)	352	55.9 (48.2, 63.3)	22.1 (17.7, 27.2)	22.0 (16.1, 29.3)	301	44.9 (37.3, 52.9)	12.4 (9.2, 16.6)	42.6 (34.9, 50.7)
Non‐Hispanic Black	382	85.4 (80.7, 89.1)	14.6 (10.9, 19.3)	205	41.5 (34.7, 48.6)^*^	39.7 (34.3, 45.3)^*^	18.9 (13.1, 26.4)	175	26.4 (18.7, 35.8)^*^	26.9 (20.0, 35.2)^*^	46.7 (38.9, 54.8)
Hispanic	816	95.3 (93.6, 96.6)^*^ ^,^ ^**^	4.7 (3.4, 6.4)^*^ ^,^ ^**^	589	42.2 (37.5, 47.0)^*^	38.4 (34.1, 42.9)^*^	19.5 (16.1, 23.3)	487	28.4 (22.8, 34.8)^*^	29.1 (23.0, 36.1)^*^	42.5 (36.6, 48.7)

Abbreviations: USDA, United States Department of Agriculture; WIC, Special Supplemental Nutrition Program for Women, Infants, and Children.

^a^ Race/ethnicity subanalyses are restricted to women who are non‐Hispanic White, non‐Hispanic Black or Hispanic.

^b^ Defined as a mother indicating any breast milk consumption in a series of questions that assessed the first thing fed, feeding in the hospital, current feeding practices and feeding from a bottle.

^c^ Defined as mother not reporting initiating breastfeeding.

^d^ Defined as infant only consuming breast milk at designated month (i.e., Month 1 or 3).

^e^ Defined as infant consuming both breast milk and infant formula at designated month (i.e., Month 1 or 3).

^f^ Defined as infant consuming infant formula only at designated month (i.e., Month 1 or 3).

^g^ Estimates do not include a response of Refused for what child is currently being fed at Month 1 (*n* = 1).

^*^ Indicates statistically significantly different than non‐Hispanic White (*t*‐test, *P* < 0.05).

^**^ Indicates statistically significantly different than non‐Hispanic Black (*t*‐test, *P* < 0.05).

Among women who intended to only feed breast milk at Month 1, less than half (45.2%) met their intention, one third (34.8%) provided both breast milk and infant formula and 19.9% provided only infant formula. A significantly lower percentage of non‐Hispanic Black women (41.5%) and Hispanic women (42.2%) met their intention of only providing breast milk at Month 1 compared with non‐Hispanic White women (55.9%, *P* < 0.05). Among women who intended to only feed breast milk at Month 3, one third (32.9%) met their intention, 24.9% provided both breast milk and infant formula and 42.2% provided only infant formula. Similar to findings for Month 1, a lower percentage of non‐Hispanic Black women (26.4%) and Hispanic women (28.4%) met their intention of providing only breast milk at Month 3 compared with non‐Hispanic White women (44.9%, *P* < 0.05).

The association of race/ethnicity with meeting prenatal breastfeeding intentions after adjusting for all the covariates is provided in Table 4. Among women who intended to try breastfeeding, Hispanic women had higher odds of meeting their intention compared with non‐Hispanic White women (adjusted odds ratios [aORs]: 4.38 [95% CI: 2.81, 6.81]). Among women who intended to feed only breast milk at Month 1, non‐Hispanic Black and Hispanic women had significantly lower odds of only feeding breast milk compared with non‐Hispanic White women after adjusting for all covariates (aORs: 0.63 [95% CI: 0.41, 0.98]; 0.64 [95% CI: 0.44, 0.92], respectively). Among women who intended to feed only breast milk at Month 3, the odds of meeting these intentions were significantly lower for both Hispanic women (aORs: 0.59 [95% CI: 0.40, 0.87]) and non‐Hispanic Black women (aOR: 0.58 [95% CI: 0.34, 0.99; *P* = 0.049]) compared with non‐Hispanic White women.

**TABLE 4:** Association of race/ethnicity^a^ with meeting breastfeeding intentions, USDA's Infant and Toddler Feeding Practices Study 2

	Meeting intention of trying to breastfeed (*n* = 1877)^b^	Meeting intention of breast milk only at Month 1 (*n* = 1226)^c^	Meeting intention of breast milk only at Month 3 (*n* = 1035)^d^
	Adjusted^e^ ^,^ ^f^ odds ratios (95% confidence intervals)		
Maternal race and ethnicity			
Non‐Hispanic White	Referent	Referent	Referent
Non‐Hispanic Black	1.43 (0.88, 2.30)	0.63 (0.41, 0.98)	0.58 (0.34, 0.99)
Hispanic	4.38 (2.81, 6.81)	0.64 (0.44, 0.92)	0.59 (0.40, 0.87)

Abbreviations: USDA, United States Department of Agriculture.

^a^ Race/ethnicity sub‐analyses are restricted to women who are non‐Hispanic White, non‐Hispanic Black or Hispanic.

^b^ Among women who strongly agreed/agreed with statement on breastfeeding intentions during pregnancy ‘I am planning to breastfeed my baby or at least try’. Meeting intention was defined as mother initiated breastfeeding.

^c^ Among women who strongly agreed/agreed with statement on breastfeeding intentions during pregnancy, ‘When my baby is one month old, I will be breastfeeding without using any formula or other milk’. Meeting intention was defined as the infant only consuming breast milk at Month 1.

^d^ Among women who strongly agreed/agreed with statement on breastfeeding intentions during pregnancy, ‘When my baby is three months old, I will be breastfeeding without using any formula or other milk’. Meeting intention was defined as the infant only consuming breast milk at Month 3.

^e^ Odds ratios are adjusted for maternal age at birth, education status, breastfeeding history, type of delivery, preterm status, presence of mother and father in same household and 2013 poverty guidelines.

^f^ Models do not include women who responded Do not Know for general breastfeeding intention (*n* = 1), for intention at 1 month (*n* = 26), and for intention at 3 months (*n* = 32).

## 4 DISCUSSION

Racial/ethnic disparities in breastfeeding rates continue to be documented, specifically non‐Hispanic Black women compared with non‐Hispanic White women (Anstey, Chen, Elam‐Evans, & Perrine, 2017; Centers for Disease Control and Prevention, 2019). We sought to understand whether or not racial/ethnic disparities may be due in part to differences in prenatal breastfeeding intentions. We found that among women in our analytic sample, nine in 10 women intended to at least try breastfeeding, six in 10 women intended to feed only breast milk at Month 1 and five in 10 women intended to feed only breast milk at Month 3. There were no differences in breastfeeding intentions at any time point between low‐income non‐Hispanic White and non‐Hispanic Black women. Hispanic women were significantly more likely than other race/ethnicities to intend to breastfeed at all time points. However, we found significant differences in whether or not a woman met her breastfeeding intentions by race/ethnicity. Hispanic women were more likely to meet their prenatal intention to initiate breastfeeding than non‐Hispanic Black and non‐Hispanic White women. Non‐Hispanic Black and Hispanic women were less likely to meet their prenatal intentions than non‐Hispanic White women for only feeding breast milk at Months 1 and 3, suggesting that non‐Hispanic Black and Hispanic women may experience additional challenges to meeting their intentions for breastfeeding duration than non‐Hispanic White women encounter.

Our findings are consistent with other research on prenatal breastfeeding intentions. For example, Langellier, Chaparro, Wang, Koleilat, and Whaley (2014) reported that 88% of WIC participants in Los Angeles County intended to breastfeed, similar to our estimate of 90% intending to at least try to breastfeed. Other studies have assessed the proportion of women meeting their individual breastfeeding goals. A population‐based cohort study in the United Kingdom found that 96% of women who intended to breastfeed for at least 4 months initiated breastfeeding (Donath et al., 2003). This may be comparable with our findings that 84–95% of women who intended to at least try breastfeeding initiated breastfeeding. However, we also saw that one in 10 women did not meet their intention to initiate breastfeeding. This may be concerning given that meeting this intention could have been accomplished by putting the baby to the breast only one time. Understanding the reasons behind this estimate are warranted but were beyond the scope of this analysis.

Research assessing longer breastfeeding duration intentions has found that not all women can breastfeed as long as they desire (Odom, Li, Scanlon, Perrine, & Grummer‐Strawn, 2013). Data from the Infant Feeding Practices Study II (IFPS II), a 2005–2007 longitudinal cohort study of US mother–infant pairs, showed that among women who intended to breastfeed for at least 2 months, 14% had stopped breastfeeding by 6 weeks (DiGirolamo, Grummer‐Strawn, & Fien, 2008). In contrast, we assessed meeting breastfeeding intentions at specific time points up to Month 3 and found high proportions of women who did not meet their intentions (e.g., 55% at Month 1). The two sample populations differ substantially and may be one of the reasons for the differences in findings. Our analyses were conducted among a low‐income population who were participating in WIC, compared with IFPS II which was conducted among a sample drawn from a consumer opinion mail panel survey in which women tended to be more educated, employed and were more likely to be White than the national population (Fein et al., 2008).

Research has consistently found that breastfeeding intentions are a strong predictor of breastfeeding behaviours (DiGirolamo, Thompson, Martorell, Fein, & Grummer‐Strawn, 2005; Donath et al., 2003; Langellier et al., 2014). However, our data suggest that women are experiencing challenges to breastfeeding in the first few weeks or months of their infant's life and that these may be more pronounced, or additional challenges are encountered, among non‐Hispanic Black and Hispanic women. There could be many reasons why women were unable to meet their breastfeeding intentions, including experiences during the birth hospitalization, access to or receipt of professional lactation services, family and community‐level support for breastfeeding, affordable childcare, and experiences within workplace environments (U.S. Department of Health and Human Services, 2011). The experience of many of these factors has been found to differ by race/ethnicity (Beauregard, Nelson, & Hamner, 2018; Evans, Labbok, & Abrahams, 2011; Jones, Power, Queenan, & Schulin, 2015; Lind, Perrine, Li, Scanlon, & Grummer‐Strawn, 2014).

Understanding the underlying factors that contribute to the differential experience of these factors for non‐Hispanic Black women and Hispanic women is an important step in helping women meet their breastfeeding intentions and reduce breastfeeding disparities. Research suggests that non‐Hispanic Black women and Hispanic women face differential treatment and expectations when it comes to breastfeeding. For example, historically, Beal et al. (2003) found that African American women were more likely to receive support for bottle feeding and less likely to receive advice on breastfeeding than White women from the WIC programme. More recently, however, African American women who breastfed reported that WIC peer counsellors or participation in a WIC peer breastfeeding support group played a positive role in helping them breastfeed (Gross et al., 2017; Mickens et al., 2009; Robinson et al., 2016). A recent review by Robinson, Fial, and Hanson (2019) recommended a more focused attention on the impacts of racism, bias and discrimination within breastfeeding research which could provide important contextual information on ways to improve breastfeeding support programs and potentially aid in the reduction of breastfeeding disparities.

There are at least two additional factors that may help explain our findings for Hispanic women. Acculturation, the process of entering a new culture in which individuals may change their attitudes and/or behaviours, has been inversely associated with breastfeeding outcomes (Ayala, Baquero, & Klinger, 2008; Lara, Gamboa, Kahramanian, Morales, & Bautista, 2005). A validated scale of acculturation was not collected in this study, limiting our ability to determine if the analytic sample was made up of more, or less, acculturated women which could have impacted our findings. Second, the concept of ‘los dos’ (‘best of both’) is the belief that providing both breast milk and infant formula provides twice the benefits for the baby (Waldrop, 2013). We found a high proportion of Hispanic women intending to breastfeed, which is consistent with other literature (Linares, Rayens, Gomez, Gokum, & Dignan, 2015; McKinney et al., 2012), but we defined meeting intentions as only providing breast milk which is in direct contrast to ‘los dos’. We found a slightly higher proportion of Hispanic women reported consuming both breast milk and infant formula than observed among non‐Hispanic White women which could suggest that ‘los dos’ is playing a role within this sample.

Our analyses were conducted among a sample of women and children enrolled in the WIC programme, which provides referrals to health care, health and nutrition education and the provision of supplemental nutritious foods to eligible low‐income pregnant, post‐partum and breastfeeding women, and children birth to 5 years of age (U.S. Department of Agriculture & Food and Nutrition Service, 2017). Specific policy‐level and programmatic efforts are included in the WIC programme to promote and support breastfeeding for participants and include prenatal and post‐natal education and promotion of breastfeeding (U.S. Department of Agriculture, 2016), tailored WIC food packages for breastfeeding mothers (U.S. Department of Agriculture & Food and Nutrition Service, 2014), an education campaign to promote breastfeeding (U.S. Department of Agriculture, 2018) and peer counselling services (U.S. Department of Agriculture, 2016). Although not all WIC participants may receive the full breadth of breastfeeding supports offered (Rasmussen et al., 2017), these services are not available to the general US population. Although we still found significant racial/ethnic differences in the ability to meet breastfeeding intentions, these data do not allow us to compare what the differences in intention or meeting intentions may have been without the support of the WIC program. Recent findings have indicated that the WIC program plays a positive role in breastfeeding initiation and duration among African American women (Gross et al., 2017; Mickens et al., 2009; Robinson et al., 2016).

This study has several strengths and limitations. First, the study design allowed us to assess prenatal breastfeeding intentions and determine whether or not women met these intentions in a prospective manner, reducing recall bias. Second, this sample consisted of low‐income women and children enrolled in WIC and had adequate sample sizes to allow for stratification by race/ethnicity, providing a unique opportunity to assess practices within a high‐risk population. However, we were unable to account for any within race/ethnicity group differences which may have impacted breastfeeding intentions and practices. Third, although our analytic sample represented 78% of women who were enrolled prenatally and therefore had responses to prenatal breastfeeding intentions, we still had missing data. Approximately 18% of those excluded (*n* = 106) had experienced a pregnancy loss and did not complete follow‐up surveys. A sensitivity analysis was conducted to assess the sociodemographic differences between those included in the analysis compared with those excluded. Results indicated that excluded women tended to be younger, have lower levels of education and were less likely to have previous breastfeeding experience. Excluding these women may have impacted our findings, but it is not clear if this would have resulted in an overestimation or an underestimation. We did not address environmental breastfeeding supports or challenges that may impact the ability to meet breastfeeding intentions. This was beyond the scope of the current analysis but is an important area for future study. Lastly, we did not have information on whether or not women changed their breastfeeding intentions after birth or if they were satisfied with the length of time they breastfed.

## 5 CONCLUSION

Among a sample of women and children enrolled in USDA's ITFPS‐2, we found no differences in prenatal intentions to breastfeed at any time point between non‐Hispanic White and non‐Hispanic Black women, but a significantly higher percentage of Hispanic women intended to breastfeed at all time points. However, non‐Hispanic Black and Hispanic women were significantly less likely than non‐Hispanic White women to meet their breastfeeding intentions at either Month 1 or 3. Based on our review of the literature and consistent with our findings, non‐Hispanic Black and Hispanic women may face additional barriers and challenges to meeting their longer breastfeeding intentions post‐partum. Efforts to identify and address these challenges may require a more thorough assessment of the underlying factors that contribute to the differential experiences non‐Hispanic Black women and Hispanic women face. Attention and inclusion of issues related to racism, bias and discrimination within breastfeeding research may be one step towards providing important contextual information to improve breastfeeding support programs and potentially aid in the reduction of breastfeeding disparities.

## CONFLICTS OF INTEREST

The authors declare that they have no conflicts of interest. The findings and conclusions in this report are those of the authors and do not necessarily represent the official position of the Centers for Disease Control and Prevention.

## CONTRIBUTIONS

HCH designed the study, conducted the analysis and drafted the initial manuscript. JLB, RL, JMN and CGP provided substantial contributions to the design of the study and interpretation of the data. All authors reviewed and revised the manuscript and approved the final manuscript as submitted.

## Footnotes

^1^ Breastfeeding initiation was assessed by a yes to any of the following questions: What was the first thing that the [child] was fed after birth?; If the first thing was other than breast milk, did you start to breastfeed your baby while still in the hospital/birthing centre?; When you left the hospital/birthing centre, were you feeding your baby only breast milk, only formula or both breast milk and formula?; Is anyone currently feeding [child] breast milk either from the breast or from the bottle, formula or both?; and Did you ever feed your baby breast milk, either from the breast or from the bottle?
